# Laparoscopic upper vaginectomy for post-hysterectomy high risk vaginal intraepithelial neoplasia and superficially invasive vaginal carcinoma

**DOI:** 10.1186/1477-7819-11-126

**Published:** 2013-06-03

**Authors:** Youn Jin Choi, Soo Young Hur, Jong Sup Park, Keun Ho Lee

**Affiliations:** 1 Department of Obstetrics & Gynecology, Seoul St. Mary’s hospital, College of Medicine, The Catholic university of Korea, 505 Banpo-dong Seocho-gu, Seoul 137-040, South Korea

**Keywords:** Vaginal carcinoma, Vaginal intraepithelial neoplasia, Vaginectomy

## Abstract

**Background:**

The aim of this study is to describe the feasibility and efficacy of the laparoscopic upper vaginectomy (LUV) in vaginal intraepithelial neoplasia(VAIN) and superficially invasive vaginal carcinoma.

**Methods:**

We studied patients with vaginal intraepithelial neoplasia (VAIN) 2, VAIN 3, and superficially invasive vaginal carcinoma after hysterectomy who have been under laparoscopic upper vaginectomy between March 2010 and March 2012.

**Results:**

Four patients underwent LUV after hysterectomy for high risk VAIN and early vaginal cancer. The mean age was 50.8 (range 40–56) years; the mean operation time was 162.5 (range 145–205) minutes; and the mean estimated blood loss was 55 (range 20–100) ml. All the patients restituted bladder function after the removal of the foley catheter. Mean hospital stay was 2 days. Two patients had postoperative complications. One patient with warfarin administration had vaginal stump bleeding and another developed vesico-vaginal fistula. Three of the patients had no residual lesion, but 1 patient had VAIN 1 in the resection margin. Colposcopy was followed on all patients and cytology proved no recurrence.

**Conclusions:**

LUV after hysterectomy is a feasible procedure and attentively applicable to high risk VAIN or superficially invasive vaginal carcinoma.

## Background

Vaginal intraepithelial neoplasia (VAIN) and vaginal carcinoma are rare clinical entities. Human papillomavirus infection, immunosuppression, radiation therapy, and smoking are reported to be the risk factors [1]. Upper vaginectomy is a technique applicable to the patients with cervical cancer after simple hysterectomy, vaginal recurrence of endometrial cancer, vaginal intraepithelial neoplasia, and superficially invasive vaginal carcinoma. The operation method has mostly been attempted via the vagina [2-4]. A few studies have reported using the laparoscopic approach, including robotically assisted laparoscopic vaginectomy [2,5,6]. However, to the best of our knowledge, there has been no study reporting laparoscopic vaginectomy in VAIN and superficially invasive vaginal carcinoma following hysterectomy. The aim of this study was to describe the feasibility and efficacy of laparoscopic vaginectomy in VAIN.

## Methods

The charts of the patients with VAIN 2, VAIN 3, and superficial vaginal carcinoma after hysterectomy, who have undergone laparoscopic vaginectomy between March 2010 and March 2012 were reviewed retrospectively. The patient details are described in Table 1.

**Table 1:** Characteristics of the patients

	**Patient 1**	**Patient 2**	**Patient 3**	**Patient 4**
Age, years	40	56	51	56
Body mass index, kg/m^2^	21.78	31.35	25.31	20.13
HPV genotype	58	56	18	Not checked
Previous hysterectomy indication	Cervical carcinoma	Cervical cancer IB	Myoma uteri	Myoma uteri
Preoperative diagnosis	VAIN 3	VAIN 2	VAIN 2	Squamous cell carcinoma
Interval between the operations, years	11	20	16	14

HPV human papilloma virus, VAIN vaginal intraepithelial neoplasia.

**Figure 1:**
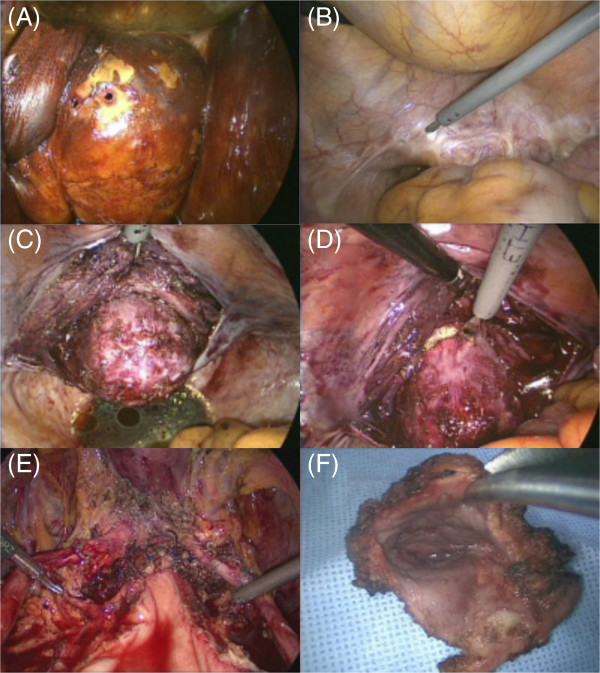
**Surgical procedures in laparoscopic upper vaginectomy.** (**A**) The planned cutting margin was identified after iodine application via a speculum examination. (**B**) Excision of the stump peritoneum. (**C**) The vaginal stump at the site of dissection of the vesicovaginal and rectovaginal spaces through the scar tissue and the isolation of the bladder pillar. (**D**) The resection of the upper vagina. (**E**) The vaginal cutting edge is restored. (**F**) The dissected upper vaginal specimen.

Colposcopy (Carl Zeiss, Inc., Berlin, Germany) was checked on all the patients to determine the resection area preoperatively. The lesions were confined to the upper one third of the vagina (Figure 1A). Under general anesthesia, the patients were placed in the Trendelenberg position and a Foley catheter was inserted. Iodine was applied on the vagina to confirm the lesion. Then, either a three-ports or single-port laparoscopic technique was performed. For the three-ports technique, one 12-mm trocar in the intraumbilicus and two 5-mm trocars in the lateral abdominal walls were used. For the single port technique, Octoport™ (Dalim surginet, Seoul, Korea) was applied. The abdomen was explored and adhesiolysis was done to secure an operation field. A rolled gauze grasped by a sponge forcep was inserted in the vagina and held with gentle upward pressure to keep adequate tension between the vagina and attendant connective tissues. After opening of visceral peritoneum over the apex of stump, the vesicovaginal and rectovaginal spaces were dissected through the scar tissue and the bladder pillar was isolated (Figure 1B and C). After the successful separation of the vaginal apex from the bladder serosa, a circumferential vaginal incision was performed and the specimen was removed (Figure 1D). The vaginal cuff was closed with a running suture using an absorbable suture (Figure 1E). The bladder was filled with normal saline to secure the dissected weak point. Supplementary pelvic lymphadenectomy was performed in the patient with vaginal cancer (patient 4). The patients were admitted until the general condition was recovered. Then, they were scheduled to visit the gynecologist’s office at 2 weeks after surgery to confirm the final diagnosis and plan further management.

## Results

The mean age was 50.8 (range 40 to 56) years. Three patients were diagnosed with high-grade VAIN (stage 2 to 3) and one with early vaginal cancer. Indications for previous hysterectomy were myoma uteri, carcinoma *in situ*, and cervical malignancy (two patients, one patient, and one patient, respectively) (Table 1). Single port laparoscopy was performed on one patient and multi-port laparoscopy was performed on the rest. The mean operation time was 162.5 (range 145 to 205) minutes and the mean estimated blood loss was 55 (range 20 to 100) ml. One patient had the Foley catheter removed on the operation day, two on the postoperative day 1, and one on postoperative day 5. All the patients restituted bladder function after the removal of the Foley catheter. They were discharged from hospital before postoperative day 3. Patient 1 had a final diagnosis of VAIN 3 and the histology confirmed VAIN 1 in the resection margin. Patient 4 had preoperative diagnosis of squamous cell carcinoma from the vaginal cytology, but the final pathological diagnosis from the vaginal stump was chronic inflammation (Table 2).

**Table 2:** Operative data of the patients

	**Patient 1**	**Patient 2**	**Patient 3**	**Patient 4**
Operation	Laparoscopic partial upper vaginectomy	Laparoscopic partial upper vaginectomy	Laparoscopic partial upper vaginectomy	Laparoscopic partial upper vaginectomy and bilateral pelvic lymphadenectomy
Operation time, minutes	150	150	145	205
Estimated blood loss, ml	20	50	50	100
Size of vaginal tissue, cm	4.2 × 4.0	3.7 × 3.2	3.1 × 2.9	3.8 × 2.8
Removal of Foley catheter, postoperative day	0	5	1	1
Hospital stay, days	3	1	2	2
Final pathology	VAIN 3 (positive resection margin with VAIN 1)	VAIN 1	Chronic inflammation	Chronic inflammation
Postoperative complications and management	Bleeding (warfarin user); gauze packing	None	Vesicovaginal fistula; indwelling catheter	None

Patient 1 had been under administration of warfarin due to mitral valve regurgitation. Warfarin was replaced by heparin for 3 days and the patient stopped the medication 1 day before the operation. On postoperative day 3, the warfarin was restarted, however, vaginal bleeding developed on postoperative day 12. The bleeding stopped after gauze packing. Patient 3 developed a vesicovaginal fistula. It was not found during the operation even after filling the bladder with blue-dyed normal saline to check the dissected weak point. Conservative management was followed; the Foley catheter was kept in place for 3 months and then the fistula was spontaneously closed. All patients were followed for 11 to 29 months and colposcopy and cytology proved no recurrence.

## Discussion

Vaginal carcinoma represents 2% to 3% of malignancy of the female genital tract and VAIN <1% of intraepithelial neoplasia [7-9]. VAIN 1 regresses spontaneously, therefore it does not require treatment [10]. For VAIN 2 and 3, no consensus on the most effective treatment has been established and various treatment modalities have been proposed, including local excision, partial or total vaginectomy, radiotherapy, laser vaporization, and topical 5-fluorouracil administration [11]. Rome *et al*. grouped 132 VAIN and early invasive vaginal cancer patients according to different treatment modalities and reported the long-term follow up results. The cure rates were 69%, 69%, and 45% when excision, laser ablation and chemical treatment were performed, respectively [3]. Radiation may be an efficacious treatment modality, however, it results in severe adverse effects, including vaginal stenosis, urinary symptoms, and vaginal ulceration [12].

There are various advantages of upper vaginectomy. First, it is a treatment modality that provides complete histopathologic information, therefore an occult malignancy is often discovered. When compared to excision, upper vaginectomy provides whole tissue, not the tissue in pieces and the lesion is removed in full depth. Moreover, vaginal lesions are multifocal and a thorough diagnostic evaluation to rule out invasive cancer is difficult; in particular in patients who have vaginal cuff with distortion post-hysterectomy, thus making follow up more challenging. Hoffman *et al*. reported to have discovered 28% of occult invasive cancer among 32 patients who underwent upper vaginectomy for VAIN 3 [4]. Indermaur *et al*. performed upper vaginectomy on 105 patients with VAIN and 12% were diagnosed with occult invasive cancer (Table 3) [7]. Moreover, occult superficially invasive vaginal carcinoma does not require adjuvant treatment, because previous studies support upper vaginectomy as an appropriate treatment [13,14]. Second, it is an effective treatment modality. Diakomanolis *et al*. reported that in high-grade VAIN, upper vaginectomy has a cure rate of 80% while laser ablation has a 68% cure rate. No recurrence was found in the current study, where others have reported recurrence of up to 21% [11]. Third, it is a safe procedure. A previous study reported that the mean estimated blood loss was 50 ml and the complication rate was 10%. The complications were cystotomy, hemorrhagy at the time of surgery, and wound cellulitis [7]. Our study resulted in 55 (20 to 100) ml of the mean estimated blood loss and one complication, a vesico-uterine fistula; postoperative bleeding in a patient with warfarin use was reported.

**Table 3:** S**ummary of cases of upper vaginectomy in VAIN 2 to 3**

**Authors**	**Number of cases**	**Procedure**	**Complication rate**	**Recurrence rate**	**Occult malignancy**
Indermaur^7^	105	Upper vaginectomy	10%	12 %	12%
Hoffman^4^	32	Upper vaginectomy	22%	16%	28%
Diakomanolis^11^	24	Upper vaginectomy	Not mentioned	21%	Not mentioned
This series	4 (including 1 vaginal cancer)	Laparoscopic upper vaginecotmy	50% (1 postopertive bleeding, 1 vesicovaginal fistula)	0%	0%

Only four cases are reported here and further studies with larger numbers of patients should be undertaken to confirm the data from this pilot study on laparoscopic vaginectomy. Moreover, long-term follow-up indicated support for laparoscopic vaginectomy as a treatment choice for VAIN and superficially invasive vaginal carcinoma.

## Conclusions

As mentioned previously, this is the first study to report LUV for post-hysterectomy VAIN and superficially invasive vaginal carcinoma by the laparoscopic approach. Laparoscopy has an advantage over the conventional approach in that the clinician can identify the distorted anatomy. The method would bring fewer complications, because the adherence of the bladder and rectum to the vault, resulting from the previous operation, is more easily dissected than in the vaginal approach. Moreover, it is an appropriate modality for patients with vaginal stenosis from prior therapy or postmenopausal vaginal atrophy. Though the study has its limitation in the small number of patients and in surgical and clinical outcomes with relatively short-term follow up, our study suggests the feasibility and efficacy of the laparoscopic vaginectomy for the post-hysterectomy patients with VAIN and superficially invasive vaginal carcinoma.

## Consent

Written informed consent was obtained from the patient for publication of this report and any accompanying images.

## Abbreviations

LUV: Laparoscopic upper vaginectomy; VAIN: Vaginal intraepithelial neoplasia.

## Competing interests

The authors declare that they have no competing interests.

## Authors’ contributions

YJC made substantial contributions to conception and design, and acquisition of data. She also drafted the manuscript and revised its final form. SYH and JSP were involved in analysis and interpretation of data. KHL contributed to interpreting the data and gave final approval of the version to be published. All authors read and approved the final manuscript.

## References

[B1] Stokes-Lampard H Wilson S Waddell C Ryan A Holder R Kehoe S Vaginal vault smears after hysterectomy for reasons other than malignancy: a systematic review of the literature BJOG 2006 11 1354 1365 10.1111/j.1471-0528.2006.01099.x 17081187

[B2] Fleisch MC Hatch KD Laparoscopic assisted parametrectomy/upper vaginectomy (LPUV)-technique, applications and results Gynecol Oncol 2005 11 420 426 10.1016/j.ygyno.2005.04.046 16005499

[B3] Rome RM England PG Management of vaginal intraepithelial neoplasia: a series of 132 cases with long-term follow-up Int J Gynecol Cancer 2000 11 382 390 10.1046/j.1525-1438.2000.010005382.x 11240702

[B4] Hoffman MS DeCesare SL Roberts WS Fiorica JV Finan MA Cavanagh D Upper vaginectomy for in situ and occult, superficially invasive carcinoma of the vagina Am J Obstet Gynecol 1992 11 30 33 10.1016/0002-9378(92)91823-S 1733213

[B5] Geisler JP Orr C Manahan KJ Robotically-assisted laparoscopic radical parametrectomy and radical vaginectomy Eur J Gynaecol Oncol 2011 11 674 676 22335034

[B6] Mahdavi A Shamshirsaz AA Peiretti M Zakashansky K Idrees MT Nezhat F Laparoscopic management of vaginal clear cell adenocarcinoma arising in pelvic endometriosis: Case report and literature review J Minim Invasive Gynecol 2006 11 237 241 10.1016/j.jmig.2006.01.011 16698533

[B7] Indermaur MD Martino MA Fiorica JV Roberts WS Hoffman MS Upper vaginectomy for the treatment of vaginal intraepithelial neoplasia Am J Obstet Gynecol 2005 11 577 580 discussion 580–571 10.1016/j.ajog.2005.03.055 16098901

[B8] Berek JS Hacker NF Berek & Hacker’s gynecologic oncology 2010 5 Philadelphia: Wolters Kluwer Health/Lippincott Williams & Wilkins

[B9] Aho M Vesterinen E Meyer B Purola E Paavonen J Natural history of vaginal intraepithelial neoplasia Cancer 1991 11 195 197 10.1002/1097-0142(19910701)68:1<195::AID-CNCR2820680135>3.0.CO;2-L 2049744

[B10] Sillman FH Fruchter RG Chen YS Camilien L Sedlis A McTigue E Vaginal intraepithelial neoplasia: risk factors for persistence, recurrence, and invasion and its management Am J Obstet Gynecol 1997 11 93 99 10.1016/S0002-9378(97)80018-X 9024096

[B11] Diakomanolis E Rodolakis A Boulgaris Z Blachos G Michalas S Treatment of vaginal intraepithelial neoplasia with laser ablation and upper vaginectomy Gynecol Obstet Invest 2002 11 17 20 10.1159/000064691 12297712

[B12] Di Saia PJ Creasman WT Clinical gynecologic oncology 2012 8 Philadelphia, PA: Elsevier/Saunders

[B13] Peters WA 3rd Kumar NB Morley GW Microinvasive carcinoma of the vagina: a distinct clinical entity? Am J Obstet Gynecol 1985 11 505 507 4061511 10.1016/0002-9378(85)90462-4

[B14] Eddy GL Singh KP Gansler TS Superficially invasive carcinoma of the vagina following treatment for cervical cancer: a report of six cases Gynecol Oncol 1990 11 376 379 10.1016/0090-8258(90)90146-C 2318447

